# Effects of stoichiometry on the transport properties of crystalline phase-change materials

**DOI:** 10.1038/srep13496

**Published:** 2015-09-03

**Authors:** Wei Zhang, Matthias Wuttig, Riccardo Mazzarello

**Affiliations:** 1 Institute of Physics (IA), RWTH Aachen University, 52056 Aachen, Germany; 2 Institute for Theoretical Solid State Physics, RWTH Aachen University, 52056 Aachen, Germany; 3 JARA-FIT and JARA-HPC, RWTH Aachen University, 52056 Aachen, Germany

## Abstract

It has recently been shown that a metal-insulator transition due to disorder occurs in the crystalline state of the GeSb_2_Te_4_ phase-change compound. The transition is triggered by the ordering of the vacancies upon thermal annealing. In this work, we investigate the localization properties of the electronic states in selected crystalline (GeTe)_x_-(Sb_2_Te_3_)_y_ compounds with varying GeTe content by large-scale density functional theory simulations. In our models, we also include excess vacancies, which are needed to account for the large carrier concentrations determined experimentally. We show that the models containing a high concentration of stoichiometric vacancies possess states at the Fermi energy localized inside vacancy clusters, as occurs for GeSb_2_Te_4_. On the other hand, the GeTe-rich models display metallic behavior, which stems from two facts: a) the tail of localized states shrinks due to the low probability of having sizable vacancy clusters, b) the excess vacancies shift the Fermi energy to the region of extended states. Hence, a stoichiometry-controlled metal-insulator transition occurs. In addition, we show that the localization properties obtained by scalar-relativistic calculations with gradient-corrected functionals are unaffected by the inclusion of spin-orbit coupling or the use of hybrid functionals.

The phenomenon of localization of electronic wave functions induced by disorder has been intensively studied in the past few decades, starting from the seminal paper by Anderson^1^. Since localized states do not contribute to transport at zero temperature, a disordered system is an insulator if the Fermi energy, *E*_*F*_, lies in the region of localized states. By changing the amount of disorder or shifting *E*_*F*_, the nature of the states at this energy can change from localized to extended, thus triggering a metal-insulator transition (MIT)^2^. More precisely, according to the celebrated scaling theory of localization^3^, this behavior occurs in three dimensions only, whereas in one-dimensional and two-dimensional systems all the electronic states are exponentially localized due to subtle quantum interference effects, irrespective of the strength of disorder, if electron correlations and spin-orbit coupling are weak.

Experimentally, a large number of studies on MITs has been carried out in doped semiconductors^4^, in particular in silicon doped with phosphorus (Si:P)^5^,^6^,^7^ and in aluminum-doped gallium arsenide (Al_x_Ga_1−x_As)^8^,^9^. In these systems, however, electron correlations are significant and the MIT is triggered by the interplay between disorder and electron-electron interactions. Recently, a MIT has been observed in the crystalline phase-change material (PCM) GeSb_2_Te_4_, which lies on the pseudobinary GeTe-Sb_2_Te_3_ line (GST). This MIT appears to be exclusively due to disorder^10^,^11^. In these experiments, at low annealing temperature, a strongly disordered rocksalt phase is obtained, wherein one of the two sublattices contains Te atoms, whereas Ge, Sb and vacancies are randomly arranged in the second one. The strength of disorder is tuned by increasing the annealing temperature. Recently, it has been shown that disorder in crystalline GST can also be tuned by pressure^12^. A disorder-driven MIT has been reported in GeTe nanowires as well^13^. In this work, the origin of insulating behavior is attributed to structural disorder, such as dislocation and antiphase boundaries, induced by external voltage pulses^13^.

Besides being of fundamental interest, these findings are important in view of possible applications in data storage. PCMs are exploited in non-volatile phase-change memories, which are based on the strong resistivity contrast between their crystalline and amorphous phase. The possibility of tuning disorder in crystalline PCMs could lead to the development of multilevel memory devices and thus to an increase in the storage density of PCM memories. In addition, disorder has been reported to have profound impact on the thermal properties of GST phase-change compounds^14^,^15^.

On the theoretical side, the study of Anderson localization and of disorder-driven MITs is very challenging, owing to the non-perturbative nature of these two phenomena (in terms of the disorder strength)^16^,^17^. Field-theoretical methods^18^,^19^,^20^, have been proven to be powerful tools to address these issues for disordered low-dimensional systems; however, a fully satisfactory description of localization in 2 and 3 dimensions is still lacking. Most computational studies of the MIT have been focused on model Hamiltonians defined on a lattice, such as the Anderson Hamiltonian^16^. Finite-size scaling methods have been developed to study the critical behavior of relevant quantities near the transition point.

First-principle studies of disordered systems based on Density Functional Theory (DFT) are very rare, since large system sizes are required to unequivocally distinguish between localized states and extended ones and obtain statistically meaningful results. Very recently, we have carried out DFT investigations^21^,^22^ of the MIT occurring in crystalline GeSb_2_Te_4_. By employing very large models of the disordered phase, we have been able to elucidate the microscopic origin of this MIT. We have shown that, in the strongly-disordered cubic phase obtained at low annealing temperature, the electronic states at *E*_*F*_ are exponentially localized inside vacancy clusters, i.e. regions where the vacancy concentration is much larger than the average value. Our simulations also indicate that these clusters dissolve and transform into ordered vacancy planes upon further thermal annealing at higher temperatures, which eventually leads to a metallic state.

The formation of a disordered rocksalt phase at low annealing temperature is a property displayed by many GST compounds^23^,^24^,^25^. Typically, this phase exhibits a large carrier (hole) concentration of the order of 10^20^ cm^−3^. For instance, the hole concentration in crystalline GeTe and GeSb_2_Te_4_ is about 5 × 10^20 ^cm^−3^ and 0.8 × 10^20^ cm^−3^, respectively, at annealing temperature of 150 °C^10^. It is believed that this property is due to the presence of non-stoichiometric, excess Ge and/or Sb vacancies^26^,^27^,^28^.

Since crystalline GeTe has been shown to be metallic even at low annealing temperatures^10^, a transition from insulating to metallic behavior must occur in crystalline GST upon increasing the GeTe content. In this work, we perform DFT simulations of the localization properties of the electronic states in selected GST compounds to investigate this phenomenon. We show that this “stoichiometry-driven” transition stems from a subtle interplay between the strong decrease in the overall number of vacancies (due to the decrease in stoichiometric vacancies) and the moderate increase in excess vacancies, the latter shifting *E*_*F*_ to the region of extended states. Our results elucidate the relationship between stoichiometry and transport properties of crystalline GST alloys.

## Results

Stoichiometric vacancies in rocksalt GeSbTe arise due to the valence mismatch between Te atoms and Ge, Sb atoms. More precisely, these vacancies ensure that the compounds have on average 3 *p*-electrons per lattice site. For instance, GeSb_2_Te_4_ has 25% stoichiometric vacancies on the cation sublattice, while in GeTe no such vacancies are needed to reach an average of 3 *p*-electrons per site. With 3 *p*-electrons per site, all PCMs should be semiconductors: their bandgap can be attributed to small distortions away from perfect octahedral arrangement (Peierls-like distortion), as well as a small degree of ionicity (charge transfer). However, experimentally, all PCMs show metallic behavior (*p*-type conductivity), after annealing to sufficiently high temperatures. This behavior is attributed to excess vacancies, which lead to an average number of *p*-electrons per atom slightly below 3. In a material like GeSb_2_Te_4_ there are apparently about 0.1-0.2% excess vacancies present, which explains the measured charge carrier concentrations. Hence, there are 25 times more stoichiometric vacancies than excess vacancies in GeSb_2_Te_4_. Both excess and stoichiometric vacancies cause disorder, if randomly distributed. On the other hand, the charge carrier concentration is only attributed to excess vacancies.

We study the compounds GeSb_2_Te_4_, Ge_3_Sb_2_Te_6_, Ge_9_Sb_2_Te_12_ and Ge_285_Sb_2_Te_288_. For each compound, we consider the crystalline (cubic or rhombohedral) phase which is formed near the crystallization temperature. We generate several models differing in the distribution of Ge, Sb and vacancies for each stoichiometry. All of these models contain 1152 sites and are constructed in an orthorhombic supercell with the (111) direction of the cubic lattice perpendicular to the XY plane of the supercell. For each compound, we include a number of excess vacancies (ranging from 1 to 4), compatible with the corresponding experimental data about carrier concentrations^10^,^29^. We employ the Perdew-Burke-Ernzerhof (PBE) functional based on the generalized gradient approximation (GGA)^30^. Calculations based on the more sophisticated hybrid functional by Heyd, Scuseria and Ernzerhof (HSE03)^31^ and spin-orbit coupling effects are discussed later on. Experimental lattice constants are used^32^,^33^. More computational details can be found in the Methods section.

### Localized electronic states

**Figure 1:**
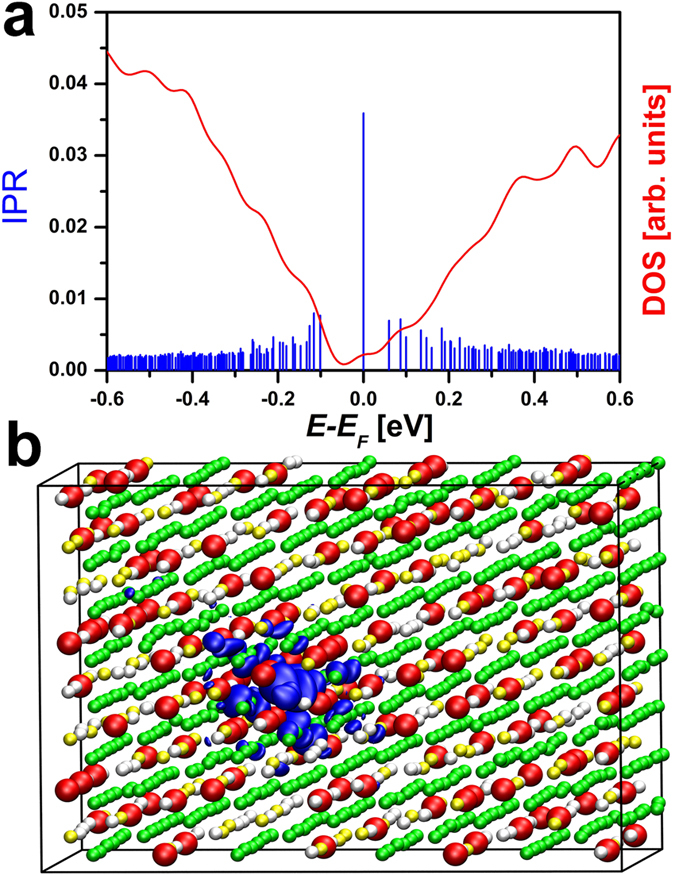
(**a**) DOS and IPR of a model of Ge_3_Sb_2_Te_6_ which contains a large vacancy cluster. (**b**) Charge density isosurface of the HOMO state of the model. The isosurface renders a value of 0.012 a.u., which roughly corresponds to the region where the exponential decay of the state occurs. Ge, Sb and Te atoms are rendered with grey, yellow and green balls, respectively. Vacancies are highlighted with bigger red balls. The state is localized within a region characterized by a large concentration of vacancies (vacancy cluster).

First, we focus on the vacancy-rich compound Ge_3_Sb_2_Te_6_. In Ref. 21, we demonstrated that clustering of vacancies leads to localization of electronic states at *E*_*F*_ in GeSb_2_Te_4_. Moreover, the presence of a realistic number of non-stoichiometric excess vacancies, corresponding to a carrier concentration of 1–2 × 10^20 ^cm^−3^, was shown not to shift *E*_*F*_ across the mobility edge *E*_*μ*_ (which separates the region of localized states from the extended ones). Here we discuss whether the same conclusion holds for Ge_3_Sb_2_Te_6_. We assume that the distribution of vacancies is uncorrelated and, therefore, we employ a random number generator (RNG) to create the models. This assumption is fully justified for disordered crystals obtained by fast crystallization of the amorphous phase^34^. Our models contain 96 stoichiometric vacancies +1–2 excess Ge vacancies. Most of the models display vacancy clusters and, in Fig. 1, we show a model which possesses a large cluster. In Fig. 1a, the density of states (DOS) and the values of the Inverse Participation Ratio (IPR) of the electronic states near *E*_*F*_ are shown. Given a state Ψ_α_, the IPR is defined as Σ_i_ |Ψ_α,i_|^4^/(Σ _i _|Ψ_α,i_|^2^)^2^, where Ψ_α,i_ indicates the expansion coefficients of Ψ_α_ with respect to the employed set of localized basis functions. The IPR characterizes the degree of localization of the electronic states. More specifically, it yields an estimate for the inverse of the number of atoms on which the state is localized. Hence, for an extended state, the IPR is equal to zero in infinitely large systems, whereas, for localized states, it remains finite.

In our model, the states deep in the valence and conduction band have IPR values of the order of 2·10^−3^. This implies that such states are completely delocalized, i.e. they spread on a number of atoms which is comparable to the total number of atoms in the supercell. On the other hand, the states in the range (−0.2, 0.2) eV around *E*_*F*_ exhibit large IPRs. In particular, the highest-occupied molecular orbital (HOMO) state at *E*_*F*_ has a very large IPR of 0.036, which indicates strong localization of the wave function. Therefore, *E*_*F*_ lies in the region of localized states and the system is insulating. This is in line with previous transport experiments^10^,^29^. The plot of a density isosurface of this state is shown in Fig. 1b. In this figure, vacancies are highlighted with red balls. The HOMO electronic wave function is well localized inside the vacancy-rich region. As occurs for Ge_1_Sb_2_Te_4_, the excess Ge vacancies do not lead to a shift of *E*_*F*_ across the mobility edge *E*_*μ*_. We stress that other similar vacancy-rich GST compounds, such as GeSb_4_Te_7_, Ge_2_Sb_2_Te_5_, Ge_4_Sb_2_Te_7_, etc., should show similar insulating behavior at low annealing temperatures, due to the same microscopic origin -- vacancy clusters.

### Ge_9_Sb_2_Te_12_

**Figure 2:**
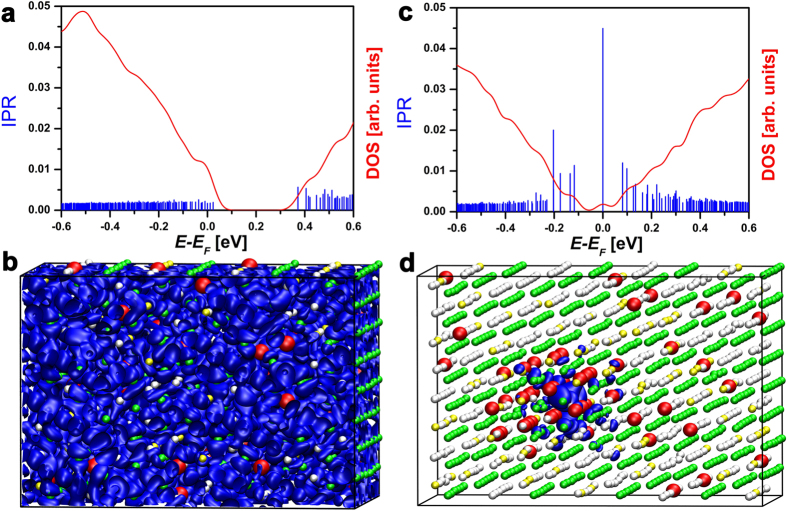
(**a**,**c**) The DOS and the IPR values of two Ge_9_Sb_2_Te_12_ models. The model on the left is generated by a RNG and does not have any vacancy cluster, while the vacancy cluster in the model on the right is generated manually. The first model has extended states at *E*_*F*_, whereas the second one has a localized HOMO state. The corresponding HOMO states of the two models are visualized in (**b,d**). The isosurfaces render a value of 0.002 and 0.012 a.u, respectively (a smaller value is used for the extended state, due to the lower charge density).

The total concentration of vacancies becomes low when the stoichiometry approaches GeTe. As a consequence, the probability to form large vacancy clusters decreases dramatically. In Ge_9_Sb_2_Te_12_, the vacancy concentration is equal to 4.2%, corresponding to 48 stoichiometric vacancies in our models. 2 additional excess vacancies are also included. Employing a RNG to construct the models, delocalized states at *E*_*F*_ are typically found. An example is shown in Fig. 2a,c. The IPR value of the HOMO state, 1.7·10^−3^, is very small. Hence, the state is extended (i.e. delocalized). Since the probability to obtain a vacancy cluster using a RNG is very low, we generate such a configuration manually. In the obtained model, the electronic states near *E*_*F*_ are well localized, see Fig. 2b,d. Notice that, on average, the energy of this model is larger (about 5 meV/atom) than that of the models generated using the RNG, which display more uniform distributions of vacancies.

Due to this energy penalty, such vacancy clusters are expected to have an even lower formation probability than that obtained by assuming uncorrelated vacancies. In fact, recent transport experiments show that the resistivity of slowly annealed Ge_8_Sb_2_Te_11_ and Ge_9_Sb_2_Te_12_ compounds does not increase significantly upon decreasing temperature down to T = 2 K. This behavior suggests that the resistivity would reach a finite value in the T = 0 K limit. Hence, these compounds are probably metallic^29^,^33^. It is important to stress that these measurements are performed on slowly annealed crystalline samples obtained starting from the as-deposited amorphous phase. Crystalline models obtained by high-temperature fast crystallization probably exhibit a higher degree of disorder.

### Extended electronic states

To gain further insights into the nature of the states in the GeTe-rich region of the pseudobinary line, we consider 1152-site models of Ge_285_Sb_2_Te_288_ containing 4 Sb atoms and 2 stoichiometric vacancies. Taking into account the high carrier concentration in GeTe^10^ (5.1 × 10^20^ cm^−3^), 4 non-stoichiometric Ge vacancies are included. The resulting total concentration of vacancies is ~0.5%. We show that none of these models exhibits localized states near the Fermi energy.

**Figure 3:**
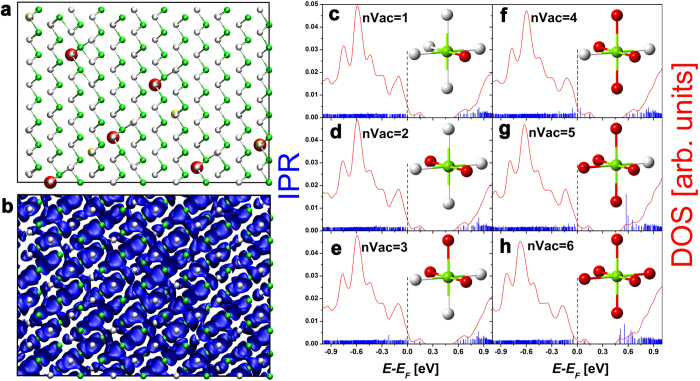
(**a**) Relaxed model of Ge_285_Sb_2_Te_288_ constructed using a RNG. The model contains six atomic vacancies. The corresponding HOMO state and the DOS/IPR values are shown in (**b**) and (**c**) respectively. The isosurface renders a value of 0.002 a.u. In (**d**)–(**h**), we show the DOS and the IPR values for models having one Te atom surrounded by 2–6 vacant neighbors. The models are generated manually. The local structural configurations (before atomic relaxation) around the relevant Te atom are shown in the insets of (**c**)–(**h**).

It is well known that GeTe forms a rhombohedral, Peierls-distorted structure at low temperatures^35^. After relaxation, an almost perfect short-long bonding pattern can be observed in our Ge_285_Sb_2_Te_288_ models, except for the regions around vacancies and Sb atoms. We plot one of the relaxed structures in Fig. 3a.

If one uses a RNG to create the models, one typically obtains sparsely distributed vacancies, which result in a few Te atoms having one nearest-neighbor vacancy (we denote this configuration as nVac = 1): see an example in the inset of Fig. 3c. Obviously, no localized electronic states are found in these models, due to the absence of vacancy clusters (see Fig. 3c for DOS and IPR values). The corresponding delocalized HOMO state is visualized in Fig. 3b. The formation of vacancy clusters is a rare event in this stoichiometry. Nevertheless, it is worth studying their effects on the electronic properties. For this purpose, we consider all the possible vacancy clusters containing up to 6 vacancies. It is particularly interesting to study configurations where a single Te atom has 2, 3 and 4 nearest neighbor vacancies (nVac = 2, 3 and 4). For the sake of completeness, we also consider the (probably unrealistic) nVac = 5 and 6 models. After full atomic relaxation, we calculate their DOS and IPR in Fig. 3e–i. Extended electronic states are found at *E*_*F*_ in all of these models, indicating metallic behavior. A few localized states are present at the edge of the conduction band in the nVac = 5 and 6 configurations. For one thing, such states are 0.5–0.6 eV above *E*_*F*_ (note that *E*_*F*_ is located in the valence band due to the presence of excess vacancies) and do not contribute to transport, for another, these low-probability configurations are energetically unfavorable (raising the total energy with respect to nVac = 1 by about 0.9 eV) and will be very easily removed upon thermal annealing. It is worth mentioning that in the relaxed nVac = 5 and 6 models, the Te atom at the center of the cluster deviates strongly from its original position and forms energetically unfavorable Te-Te bonds (more details are provided in the supplementary information). This behavior originates from the fact that Te possesses 6 valence electrons and needs to form additional bonds to fill the open *p* shell. In the supplementary information, we also discuss stoichiometric GeTe-rich compounds containing nVac = 5 and 6 clusters. It turns out that, for stoichiometric models, these configurations can induce localization of the HOMO state. However, the latter models do not include a crucial ingredient, namely excess vacancies, and are thus not relevant to current experiments. Nevertheless, they suggest that Anderson localization phenomena could, in principle, be observed even in GeTe-rich GST compounds, if one would be able to reduce self-doping effects.

In principle, vacancies can form larger clusters in this stoichiometry than those considered here (the study of which would require bigger models), however the probability of such clusters is exceedingly small. Furthermore, these clusters are also expected to induce localization only at energies well above *E*_*F*_, due to the presence of a significant number of excess vacancies. Therefore, we conclude that GeTe-rich GST compounds display metallic behavior, owing to the extended nature of the electronic states near *E*_*F*_. Similar conclusions are expected to hold for GeTe, in the presence of excess vacancies.

### Hybrid functional corrections

**Figure 4:**
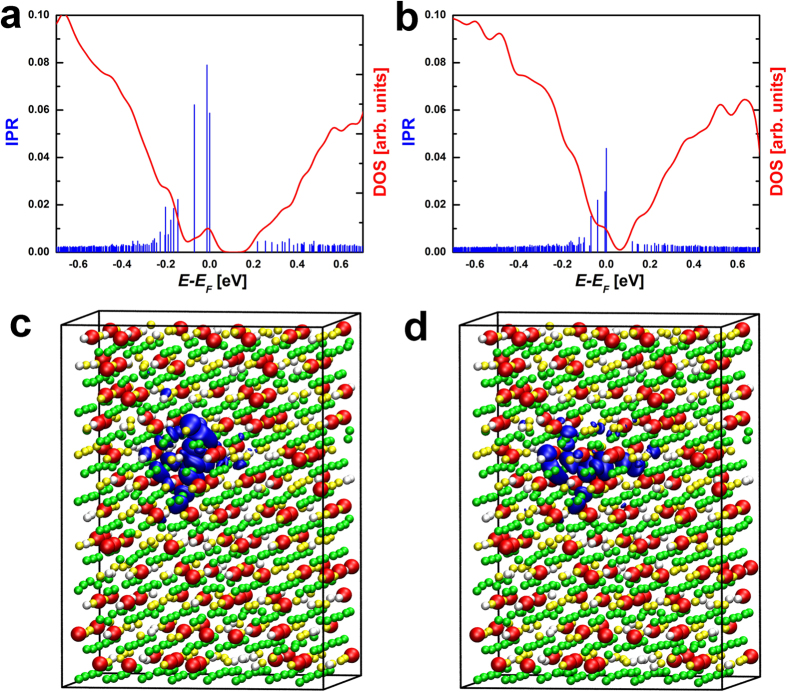
IPR and DOS of a model of cubic GeSb_2_Te_4_ calculated with (a) the HSE03 functional and (b) the GGA-PBE functional. The corresponding HOMO states are shown in (**c**) and (**d**). The isosurfaces render a value of 0.012 a.u.

Standard local-density-approximation (LDA) functionals and GGA functionals can lead to underestimation of charge fluctuations, due to self-interaction effects, thereby inducing a spurious increase of the localization length of electronic states^36^. The fact that our GGA calculations yield localized states in our disordered models of vacancy-rich GST alloys makes us completely confident that strong localization occurs in these compounds. Nonetheless, it is interesting to perform test calculations using hybrid functionals, which can partially cure self-interaction effects, and compare them with GGA results. For this purpose, we calculate the electronic properties of the model of disordered cubic GeSb_2_Te_4_ shown in Fig. 3 of Ref. 21 (where it is denoted as Cub-25%) employing the HSE03 functional^31^. We also compute the IPR of the wave functions near *E*_*F*_. Not surprisingly, the states near *E*_*F*_ are even more localized (inside the same vacancy cluster) if hybrid functionals are employed. In particular, the IPR of the HOMO state is larger, 0.059 (HSE03) versus 0.044 (GGA), as shown in Fig. 4. As regards the band structure, the most important effect due the hybrid functional is the increase in the band gap size, from 0.12 (GGA) to 0.23 eV (HSE03). As far as the metallic side of the MIT is concerned, hybrid functional simulations of our models of GeTe-rich alloys, such as Ge_285_Sb_2_Te_288,_ clearly indicate complete delocalization of the states at *E*_*F*_, in agreement with GGA results.

### Spin-orbit coupling effects

**Figure 5:**
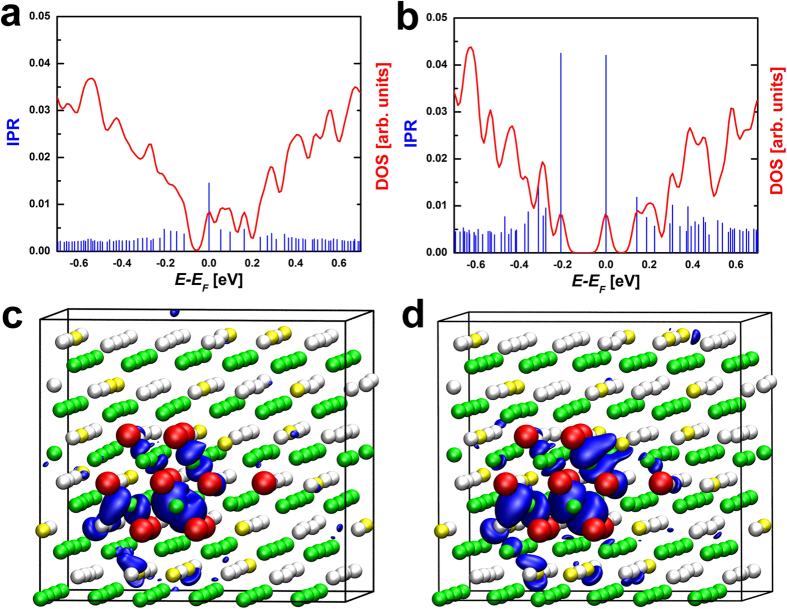
IPR and DOS of a cubic model of Ge_9_Sb_2_Te_12_ calculated (a) with and (b) without SOC. The calculations are carried out using Quantum Espresso^49^. The corresponding HOMO states are shown in (**c**) and (**d**). The isosurfaces render a value of 0.012 a.u.

Next, we investigate the SOC effects on the localization properties. This interaction is known to be strong in GST compounds and can affect the electronic structure and transport properties significantly^37^,^38^,^39^. Due to the very high computational cost of SOC calculations, it is unfeasible to investigate 1152-site models. Hence, we study a smaller, 276 atom model of Ge_9_Sb_2_Te_12_ containing 12 vacancies. No excess vacancies are considered because of the small size of the model. More precisely, the number of carriers produced by one Ge excess vacancy in this model would already be 2–3 times larger than the experimental one. We arrange these stoichiometric vacancies close together so as to obtain a large cluster, which induces strong localization of the HOMO state (see Fig. 5). The localization length of this state is smaller than the system size. Upon inclusion of SOC, the HOMO state remains localized and its shape is largely unaffected. The change in the IPR value, 0.042 vs 0.015, is due to the change in the basis set (the IPR of a state depends on the localized basis set onto which the state is projected; see also Methods section). Note that the IPR values for the delocalized states are also reduced. Interestingly, the band gap decreases from 0.14 eV to 0.06 eV due to SOC effects.

## Discussion

**Figure 6:**
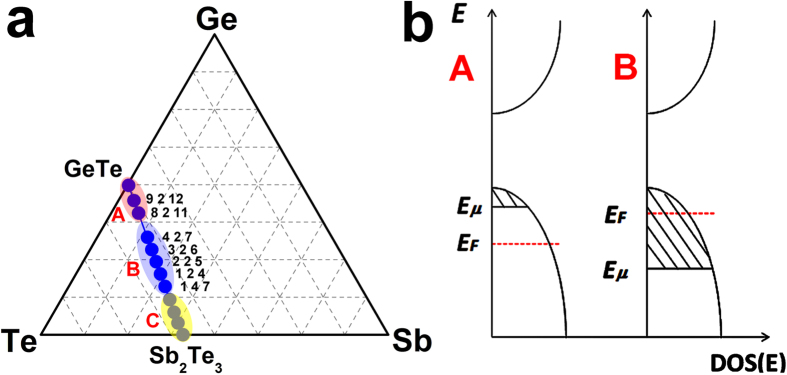
(**a**) The pseudo-binary GeTe-Sb_2_Te_3_ line can be divided into three regions, which are characterized by different transport properties of the alloys. In region A, GST alloys (including GeTe) are found to be metallic at any annealing temperature due to 1) the absence of sizable vacancy clusters (owing to the relatively small number of vacancies) and 2) self-doping effects (i.e. the presence of excess vacancies). The corresponding DOS are schematically depicted in (**b**), where the shaded regions indicate localized states. In region B, GST compounds display insulating behavior at low annealing temperatures, due to the presence of vacancy clusters which lead to a relatively wide energy region containing localized states (**b**), and undergo a transition to metallic behavior upon thermal annealing at high temperatures. In region C, GST alloys (including Sb_2_Te_3_) do not form cubic phases under normal conditions and exhibit metallic behavior due to similar reasons as those given for region A.

We summarize our findings on the localization properties of (GeTe)_x_-(Sb_2_Te_3_)_y_ compounds belonging to the Ge-Sb-Te ternary diagram in Fig. 6. We have shown that, in GeTe and GeTe-rich compounds (indicated with A in the figure), the total concentration of vacancies is small and, therefore, the probability of forming sizable clusters is also small, leading to a very narrow region of localized states. Moreover, the number of excess vacancies is large enough to move *E*_*F*_ to the region of extended states, as schematically depicted in the inset of Fig. 6, where the shaded regions denote localized states. As a result, these compounds are metallic.

On the other hand, in vacancy-rich GST compounds (region B), states at the Fermi energy are localized inside vacancy clusters. The abundance of vacancies makes the formation of such clusters likely, resulting in a wide region of localized states, while the concentration of excess vacancies is not sufficient to shift the Fermi energy across the mobility edge *E*_*μ*_ (see inset of Fig. 6). Hence, *E*_*F*_ lies in the region of localized states and the system exhibits insulating behavior.

A metal-insulator transition occurs upon thermal annealing^10^,^11^,^21^ in vacancy-rich GST compounds at fixed stoichiometry, such as GeSb_2_Te_4_ and Ge_3_Sb_2_Te_6_. Our results show that another metal-insulator transition takes place in crystalline GST at fixed (low) annealing temperature upon changes in stoichiometry from vacancy-rich to GeTe-rich (vacancy-poor) compounds. Hence, the stoichiometry can also be employed to tune the transport properties of GST alloys.

Our investigation of the effects due to spin-orbit coupling and hybrid functionals shows that the above findings are robust and are not artifacts of the approximations employed (semi-local functionals and scalar-relativistic approximation).

If one approaches the other end of the pseudo-binary line, Sb_2_Te_3_ (region C in Fig. 6), it becomes increasingly difficult to obtain a cubic phase experimentally. It has been found that Sb_2_Te_3_-rich compounds exhibit stable hexagonal structures with very elongated primitive cells: for instance, GeSb_6_Te_10_ displays a 51-layer periodicity along the c direction^40^,^41^. No rocksalt-like structure has been reported in these experimental works. Under normal conditions, Sb_2_Te_3_ forms a trigonal, layered structure with a periodicity of 21 layers and is known to be a topological insulator^42^,^43^; nevertheless, due to self-doping effects, Sb_2_Te_3_ typically shows *p*-type metallic behavior as well. Hence, a second insulator-metal transition is expected to occur in the Sb_2_Te_3_-rich region of the pseudobinary line. Further experiments are needed to explore the structural and transport properties of GST compounds in this stoichiometry range.

In this work, we have assumed that electron-electron interaction effects do not play an important role in GeSbTe compounds and the MITs observed upon changes in stoichiometry (or annealing temperature) are solely due to disorder. This assumption is based on solid experimental facts, namely: a) correlations are weak due to the large dielectric constants and, thus, effective screening effects and b) disorder is strong owing to the large concentration of vacancies. In fact, critical carrier concentrations in GeSbTe are far too large to satisfy Mott’s criterion for a correlation-induced MIT^10^. Furthermore, MIT transitions in GeSbTe are not driven by changes in carrier concentrations. On the contrary, this quantity does not vary strongly as a function of annealing temperature or stoichiometry. These properties make GeSbTe compounds fundamentally different from doped semiconductors such as Si:P^44^,^45^, where the transition is due to the concerted effect of disorder and correlation. In Si:P, impurities contribute to both disorder and carrier concentration. In PCMs, on the other hand, stoichiometric vacancies only contribute to disorder, whereas the carrier concentration is controlled by the excess vacancies. Varying the stoichiometry along the pseudobinary GeTe-Sb_2_Te_3_ line or changing the annealing temperature mostly affects the concentration or arrangement of stoichiometric vacancies and, thus, the degree of disorder. Hence, these materials provide us with the opportunity to investigate a purely disorder-driven MIT. This fact also justifies the use of DFT methods to study these materials: standard DFT functionals are insufficiently accurate to grasp the subtle interaction effects responsible for Mott MITs but can properly describe disorder-induced localization effects.

## Methods

Most of the calculations are carried out with the QUICKSTEP code^46^ included in the CP2K package^47^. This DFT code is based on a mixed Gaussian and plane-wave approach. Kohn-Sham orbitals are expanded in a Gaussian-type basis set of triple-zeta plus polarization quality, whereas the charge density is expanded in plane waves, with a cutoff of 300 Ry. Scalar-relativistic Goedecker^48^ pseudopotentials and GGA-PBE functionals^30^ are used. The Brillouin zone is sampled at the *Γ* point only. Spin-orbit coupling calculations are performed using the Quantum Espresso package^49^. We employ fully-relativistic ultrasoft pseudopotentials^50^ and PBE functionals^30^. These calculations are also carried out within the *Γ* point approximation.

We use different sets of localized basis functions to calculate the IPR values of the electronic states. In the case of CP2K, since wave functions are expanded in a Gaussian-type basis set, the same localized functions are employed to compute the IPR. On the contrary, in Quantum Espresso the Bloch states are expanded in a plane-wave set, and the IPR values are obtained by calculating the projections of the Kohn-Sham states onto atomic-like orbitals. The use of two different sets of localized basis functions results in different IPR values. Furthermore, the IPR values obtained from Quantum Espresso calculations with and without spin-orbit interaction also differ, due to the use of different sets of atomic-like orbitals.

## Additional Information

**How to cite this article**: Zhang, W. *et al.* Effects of stoichiometry on the transport properties of crystalline phase-change materials. *Sci. Rep.* **5**, 13496; doi: 10.1038/srep13496 (2015).

## Supplementary Material

Supplementary Information
