# Supplementary material for: Effects of stoichiometry on the transport properties of crystalline phase-change materials
Source: Sci Rep. 2015 Sep 3;5:13496. doi: 10.1038/srep13496 (PMC4558572; doi:10.1038/srep13496)
Supplement: Supplementary Information [file srep13496-s1.pdf]

Supplementary Information

Effects of stoichiometry on the transport properties of crystalline phase-change materials

Wei Zhang[1,2], Matthias Wuttig[1,3] and Riccardo Mazzarello[2,3]*

[1] I. Institute of Physics (IA), RWTH Aachen University, 52056 Aachen, Germany

[2] Institute for Theoretical Solid State Physics, RWTH Aachen University, 52056 Aachen, Germany

[3] JARA-FIT and JARA-HPC, RWTH Aachen University, 52056 Aachen, Germany

* Corresponding author. Email address: mazzarello@physik.rwth-aachen.de

In this supplement, we consider GeTe-rich GeSbTe compounds and discuss low probability configurations where one Te atom has 5 or 6 vacant neighbors. Both stoichiometric and non-stoichiometric (i.e. with excess vacancies) GeSbTe compounds are considered.

Model of $Ge_{566}Sb_4Te_{576}$ containing 2 stoichiometric and 4 excess vacancies.

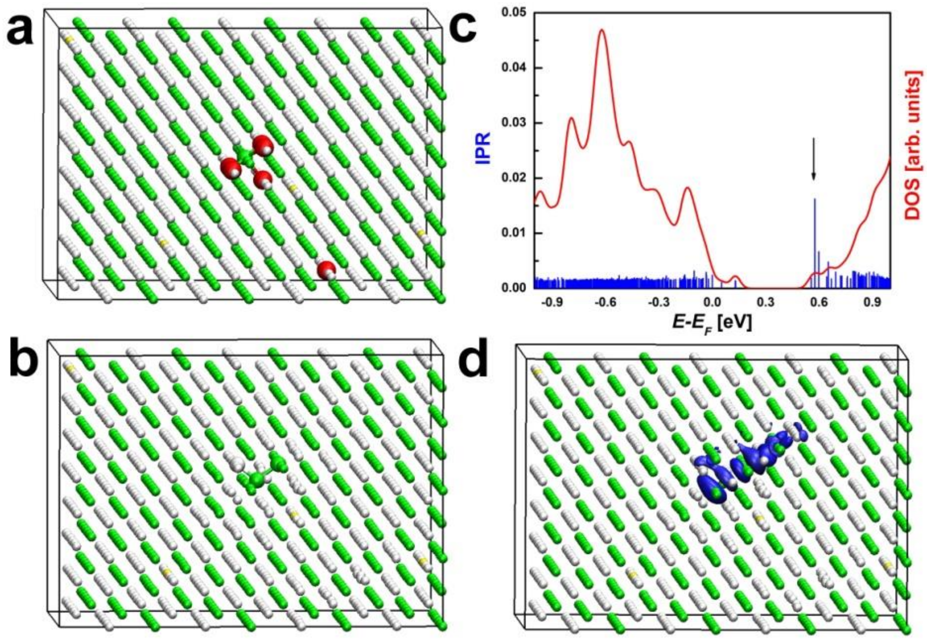

Figure S1: Model of $Ge_{566}Sb_4Te_{576}$ containing a Te atom with 5 nearest neighbor vacancies. **a** and **b** show the unrelaxed and relaxed geometry of the model. The Te atom relaxes from its initial position and forms an additional Te-Te bond (in the unrelaxed structure, it only forms a Ge-Te bond). The inverse participation ratio (IPR) and density of states (DOS) are shown in **c**. At 0.6 eV above the Fermi energy, a few localized states exist. The empty state marked by the arrow in **c** is visualized in **d**. The isosurface render a value of 0.012 a.u.

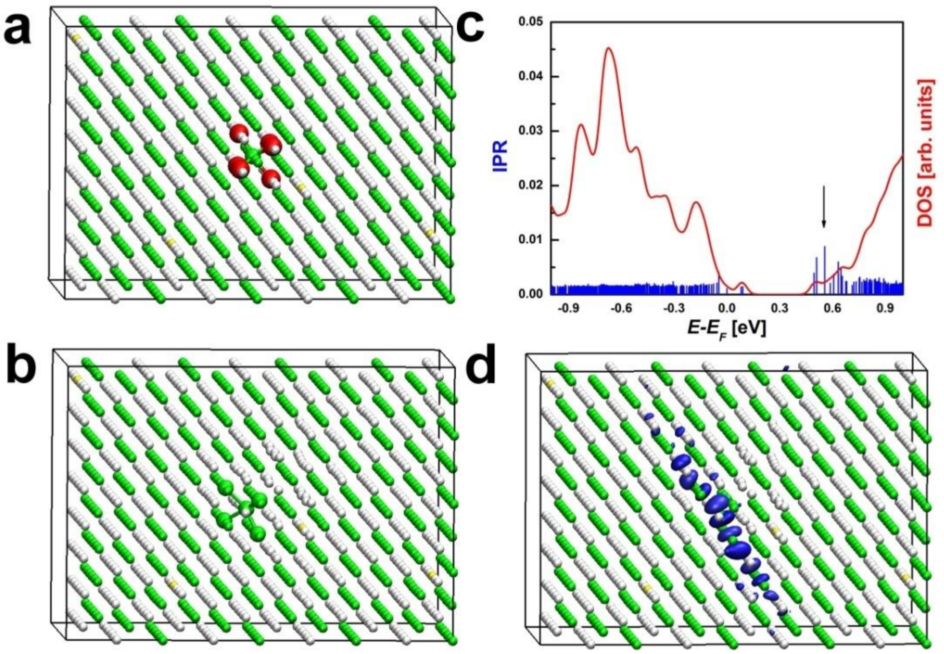

Figure S2: Model of $Ge_{566}Sb_4Te_{576}$ containing a Te atom with 6 nearest neighbor vacancies. **a** and **b** show the unrelaxed and relaxed geometry of the model. The Te atom relaxes from its initial position and occupies a site of the second sublattice, thus forming an anti-site defect. As a result, several Te-Te bonds are formed. The IPR and DOS are shown in **c**. At 0.5-0.6 eV above the Fermi energy, several localized states exist. The empty state marked by the arrow in **c** is visualized in **d**. The isosurface renders a value of 0.012 a.u.

Model of $Ge_{558}Sb_{12}Te_{576}$ containing 6 stoichiometric vacancies.

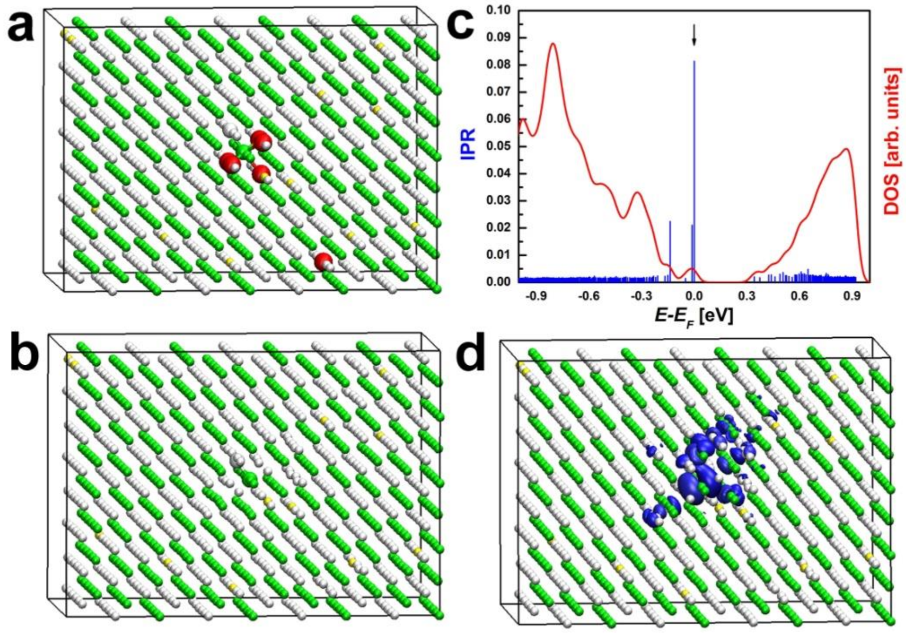

Figure S3: Model of $Ge_{558}Sb_{12}Te_{576}$ containing a Te atom with 5 nearest neighbor vacancies. **a** and **b** show the unrelaxed and relaxed geometry of the model. The IPR and DOS are shown in **c**. A few localized states exist at

the edge of the valence band: in particular, the HOMO state is well localized. The HOMO state is visualized in **d**. The isosurface renders a value of 0.012 a.u.

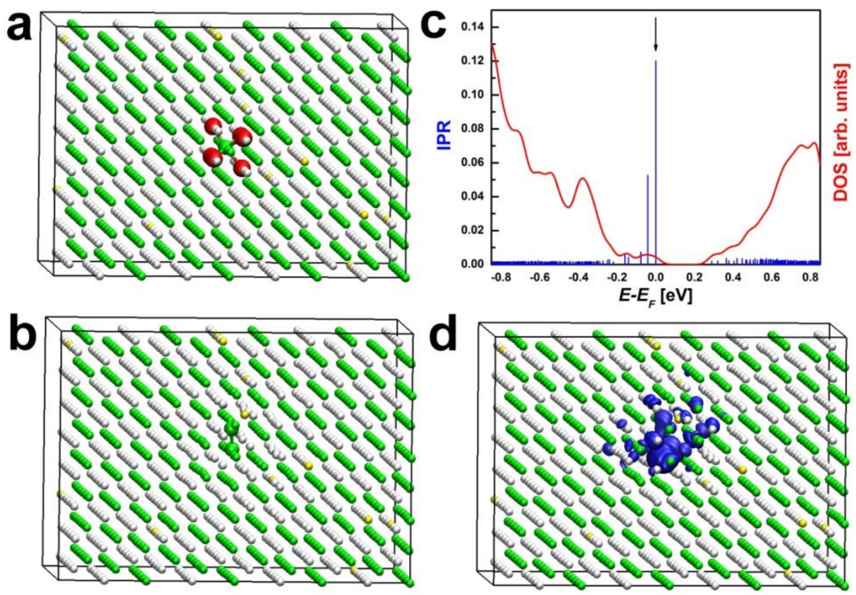

Figure S4: Model of $Ge_{558}Sb_{12}Te_{576}$ containing a Te atom with 6 nearest neighbor vacancies. **a** and **b** are the unrelaxed and relaxed geometries of the model. The Te atom and a nearby Te atom move towards each other and form a Te-Te bond. The IPR and DOS are shown in **c**. A few localized states exist at the edge of the valence band: in particular, the HOMO state is well localized. The HOMO state is visualized in **d**. The isosurface renders a value of 0.012 a.u.
